# Genetic link between *KIF1A* mutations and amyotrophic lateral sclerosis: evidence from whole-exome sequencing

**DOI:** 10.3389/fnagi.2024.1421841

**Published:** 2024-07-15

**Authors:** Wei Zheng, Ji He, Lu Chen, Weiyi Yu, Nan Zhang, Xiaoxuan Liu, Dongsheng Fan

**Affiliations:** ^1^Department of Neurology, Peking University Third Hospital, Beijing, China; ^2^Beijing Key Laboratory of Biomarker and Translational Research in Neurodegenerative Diseases, Beijing, China; ^3^Key Laboratory for Neuroscience, National Health Commission/Ministry of Education, Peking University, Beijing, China; ^4^Biomedical Pioneering Innovation Center (BIOPIC), Peking University, Beijing, China

**Keywords:** amyotrophic lateral sclerosis, *KIF1A*, axonal transport, *KIF5A*, cosegregation analysis

## Abstract

**Objectives:**

Genetics have been shown to have a substantial impact on amyotrophic lateral sclerosis (ALS). The ALS process involves defects in axonal transport and cytoskeletal dynamics. It has been identified that *KIF1A*, responsible for encoding a kinesin-3 motor protein that carries synaptic vesicles, is considered a genetic predisposing factor for ALS.

**Methods:**

The analysis of whole-exome sequencing data from 1,068 patients was conducted to examine the genetic link between ALS and *KIF1A*. For patients with *KIF1A* gene mutations and a family history, we extended the analysis to their families and reanalyzed them using Sanger sequencing for cosegregation analysis.

**Results:**

In our cohort, the *KIF1A* mutation frequency was 1.31% (14/1,068). Thirteen nonsynonymous variants were detected in 14 ALS patients. Consistent with the connection between *KIF1A* and ALS, the missense mutation p.A1083T (c.3247G>A) was shown to cosegregate with disease. The mutations related to ALS in our study were primarily located in the cargo-binding region at the C-terminal, as opposed to the mutations of motor domain at the N-terminal of *KIF1A* which were linked to hereditary peripheral neuropathy and spastic paraplegia. We observed high clinical heterogeneity in ALS patients with missense mutations in the *KIF1A* gene. *KIF5A* is a more frequent determinant of ALS in the European population, while *KIF1A* accounts for a similar proportion of ALS in both the European and Chinese populations.

**Conclusion:**

Our investigation revealed that mutations in the C-terminus of *KIF1A* could increase the risk of ALS, support the pathogenic role of *KIF1A* in ALS and expand the phenotypic and genetic spectrum of *KIF1A*-related ALS.

## Introduction

Amyotrophic lateral sclerosis (ALS) is a debilitating neurodegenerative disorder with an average life expectancy of merely 2–5 years following diagnosis (Brown and Al-Chalabi, 2017; Feldman et al., 2022). This disease presents itself as a degeneration of the limbs in spinal-onset ALS or challenges with speaking and/or swallowing in bulbar-onset ALS; gradual muscle weakness emerges, leading to death from respiratory failure in the end (Goutman et al., 2022b). The intricate pathophysiology of ALS remains incompletely understood. Genetics play a pivotal role in this phenomenon, and ALS can be hereditary, with familial ALS accounting for 15% of cases and the other 85% being classified as sporadic ALS (sALS) (Goutman et al., 2022a). At least 40 genes have been associated with ALS, providing significant insights into its pathophysiology (Chia et al., 2018; Brenner and Freischmidt, 2022). Nevertheless, the exact way in which ALS genes play a role in the development of ALS is yet to be determined.

Intracellular transport plays a vital role in maintaining the function, morphogenesis, and homeostasis of neurons. This is because neurons produce the majority of proteins necessary for axon and nerve terminal activities in the cell body, which then need to be transported to precise locations (Hirokawa et al., 2009). Numerous genetic, pathological, and neurobiological findings have established that axonal transport deficits act a significant role in the progression of ALS (Bilsland et al., 2010; Castellanos-Montiel et al., 2020). For example, numerous genes associated with ALS, including *DCTN1*, *KIF5A*, *ALS2*, *NEFH*, *PFN1*, and *SPAST*, participate in controlling cytoskeletal dynamics and function and regulate intracellular transport events (Liu et al., 2017; Nicolas et al., 2018; Castellanos-Montiel et al., 2020). In the process of ALS, the beginning phases entail the deterioration of extended axons in motor nerve cells, beginning at the farthest locations and advancing in a pattern known as “dying back” (Baldwin et al., 2016; Guo et al., 2017). Additionally, axonal transport deficits precede ALS symptoms; therefore, axonal transport could be an indicator of motor neuron degeneration (Fischer et al., 2004; Bilsland et al., 2010).

Kinesin superfamily proteins (KIFs) are a group of main molecular motors that responsible for transporting cargoes, including proteins, membranous organelles, and mRNAs, toward axon terminals along microtubules (Hirokawa et al., 2009). *KIF1A* encodes a molecular motor of the kinesin-3 variety, which is responsible for transporting synaptic vesicles, dense core vesicles, precursors of synaptic vesicles, and precursors of the active zone (Edwards et al., 2015; Guedes-Dias et al., 2019). Dense core vesicles mainly contain neurotransmitters and neuropeptides, while synaptic vesicle precursors and synaptic vesicles mainly transport VAMP2, RAB3A, and synaptophysin (Stucchi et al., 2018). *KIF1A* deficiency leads to remarkable impairments in motor and sensory functions, reduced density of synaptic vesicles at nerve terminals, and the buildup of transparent vesicles in neuronal cell bodies (Tanaka et al., 2016; Anazawa et al., 2022). To date, three *KIF1A*-associated disorders have been included in the OMIM classification: spastic paraplegia type 30 (SPG30, #610357), with recessive inheritance; and NESCAV syndrome (#614255), with dominant inheritance, and hereditary peripheral neuropathy, refer to hereditary sensory and autonomic neuropathy type 2 (HSAN2, #614213) (Nicita et al., 2021). Recently, *KIF1A* was recognized as a novel causative gene for ALS in the southern Chinese population (Liao et al., 2022). However, there is a lack of evidence indicating that *KIF1A* is a genetic risk factor for ALS. Therefore, we detected rare *KIF1A* variants in 1,068 ALS patients, comprising 988 sporadic and 80 familial cases, using whole-exome sequencing (WES), and analyzed the genotype–phenotype relationship in patients with *KIF1A* variants, deepening our understanding of how *KIF1A* deficiency affects ALS pathogenesis.

## Materials and methods

### Participants and data collection

A total of 1,068 Chinese ALS patients, 988 with sporadic ALS and 80 unrelated individuals with familial ALS, were enrolled at Peking University Third Hospital (PUTH) from 2007 to 2023. This cohort of ALS patients included 677 individuals, who underwent DNA extraction and exome sequencing in a previous study by our team (Liu et al., 2021). Patients diagnosed with probable or definite ALS based on the Airlie House diagnostic criteria were included in the study (Brooks et al., 2000). A total of 1,812 healthy controls, who had no previous neurological impairment, were also enrolled in the study. The exclusion criteria for both patients and controls was the unavailability of DNA sample. All patients and controls were of Han ethnicity. Baseline clinical data and demographic information, including age, sex, family history, smoking and drinking history, age at onset, location of initial symptoms, diagnostic delay, King’s college staging system, and revised ALS functional rating scale scores, were collected during each patient’s first visit to PUTH. The Edinburgh Cognitive and Behavioral Assessment Screen was used to assess patient cognition. Patients were followed up with by neurologists through in-person visits or by telephone every 3 months. The Institutional Ethics Committee of PUTH approved this study (IRB00006761), and all individuals involved gave their written informed consent.

### DNA extraction

Samples of blood were collected from both patients and healthy volunteers. DNA was isolated from periphery venous blood. DNA extraction was performed using QIAmp DNA blood Mini Kit (QIAGEN, Hilden, Germany).

### WES analysis

WES was used to screen all subjects, including both patients and healthy controls. Genomic DNA (1 μg) was fragmented into 200–300 base pair lengths using a Covaris Acoustic System. These DNA fragments underwent a series of processes including end repair, A-tailing, and adaptor ligation. Subsequently, a 4-cycle precapture polymerase chain reaction (PCR) amplification and a targeted sequence capture were performed. Postcapture, the DNA fragments were eluted and amplified through 15 cycles of PCR. The final sequencing products were read in 150 bp paired-end mode on the Illumina HiSeq X platform in accordance with the standard protocol. Using BWA 0.5.9,^1^ pair-ended reads were mapped to the hg19/GRCh37 version of the human genome reference. To identify single nucleotide variants and small insertions and deletions (INDELs), Genome Analysis Toolkit (GATK) was employed^2^ (McKenna et al., 2010). All suspected variants were validated by Sanger sequencing. Additional classical pathogenic ALS-related genes, including *SOD1*, *SETX*, *FUS*, *ALS2*, *OPTN*, *TARDBP*, *DCTN1*, *VAPB*, *Fig 4*, *TBK1*, *CHCHD10*, *ANXA11*, *NEK1*, and *SQSTM1* were examined by WES analysis. The length of *C9orf72* repeat alleles was evaluated using a two-step PCR method involving fluorescent fragment-length analysis followed by repeat-primed PCR, as described in previous studies (Tang et al., 2022).

### Quality control (QC)

We conducted the QC procedures to filter out genetic variants through the following criteria: (1) genotype call rate under 99%; (2) Hardy–Weinberg equilibrium deviation in control groups (*p* < 10E-6); (3) significant missingness discrepancies between cases and controls (*p* < 10E-6); and (4) presence of three or more alleles. Variants that did not meet the quality control criteria were discarded.

### Filtering of damaging mutations

*KIF1A* variants that met the following criteria were selected for further analysis: nonsynonymous, indel or putative splice site mutations; minor allele frequency (MAF) ≤ 0.1% for heterozygous variants; and MAF less than 1% in the Exome Aggregation Consortium (ExAC) and Genome Aggregation Database (gnomAD) databases. The pathogenicity of the identified variants was evaluated following American College of Medical Genetics and Genomics (ACMG) guidelines. To evaluate the potential functional outcomes of each variant, we employed eight bioinformatic tools designed to predict the potential effects of a substitution in the amino acid on the structure and established function of a human protein: MutationTaster,^3^ SIFT,^4^ PolyPhen-2,^5^ MetaLR, MetaSVM, ClinPred,^6^ M_CAP,^7^ and CADD.^8^

### Sanger sequencing

PCR was conducted in a total volume of 25 μL, comprised of genomic DNA, primers, and 2 × Taq PCR Master Mix (Tsingke Biotechnology Co., Ltd., Beijing, China). The PCR reaction parameters were the following: pre-heating at 94°C for 5 min, denaturing at 94°C for 30 s, annealing at 55°C for 30 s, extending at 72°C for 30 s, with a total of 35 cycles, ending with a final extension at 72°C for 5 min, followed by cooling to 4°C. Subsequently, the PCR samples underwent sequencing utilizing the Sanger Chain Termination technique.

### Cosegregation analysis for families

Genomic DNA extracted from blood samples from patient families underwent genomic analysis. PCR was employed to screen all individuals for *KIF1A* sequences, encompassing the same region examined in the proband. To facilitate the amplification process, forward (CAGGGCCTCACTTGAACTGG) and reverse primers (AAGAGCTTCGCATCGTGGAG) were used. Using the CodonCode Aligner software, the DNA sequences obtained from the samples were compared and matched with the UCSC hg19 reference human genome.

### Statistical analysis

Descriptive statistics (means ± SDs) were calculated for continuous variables. Statistical analysis was conducted using GraphPad Prism version 8.4.0.

## Results

### Mutation analysis of the *KIF1A* gene

We analyzed the *KIF1A* sequence in a group of 1,068 ALS patients. The demographic and clinical features of the ALS patients are presented in Table 1. Thirteen nonsynonymous variants were detected in 14 ALS patients, all of which were heterozygous. In our cohort, the *KIF1A* mutation frequency was 1.31% (14/1,068). Among the 13 variants, 12 had missense variations, while one had a delete-insert mutation. Except for the variant p.A918deinsGA (c.2753_2754insGGA, P2), the other 12 variants had a <0.1% allele frequency in the gnomAD and ExAC databases (Table 2). Additionally, p.P424 L (c.1271C>T, P1) and p.P1178S (c.3533 T>C, P6) were not reported in any of the databases. The functional predictions revealed that 11 missense variants were predicted pathogenic at least one silico tool based on eight silico tools totally (Table 2). Four variants [p.E979K (c.2935G>A, P3), p.V1255M (c.3763G>A, P8), p.D1711N (c.5131G>A, P13), and p.R1717L (c.5150G>T, P14)] were predicted pathogenic through 5–6 silico tools. Four variants were identified as likely pathogenic (LP) according to the ACMG standards and guidelines (Table 2): p.V1255M (c.3763G>A, P8), p.P1593L (c.4778C>T, P10), p.D1643N (c.4927G>A, P11), and p.R1717L (c.5150G>T, P14). The ACMG evidence for the pathogenicity of these variants was strong, and they were predicted to be damaging by bioinformatic tools. Among them, p.D1643N (c.4927G>A, P11) and p.R1717L (c.5150G>T, P14) were reported previously in Human Gene Mutation Database (HGMD) (Table 2). Additionally, other nine variants were uncertain significance (VUS) according to the ACMG standards. In total, 11 variants were recognized novel variants in ALS patients. The detailed variant information is listed in Table 2.

**Table 1:** Demographic and clinical characteristics of ALS patients.

Variables	ALS	Control
Number of patients	1,068	1,812
Sex ratio (male/female)	1.6 (657/411)	1.0 (901/911)
Age at onset (years)	51.3 ± 11.2	–
Sporadic/familial	998/80	–

ALS, amyotrophic lateral sclerosis.

**Table 2:** Overview of variants in the *KIF1A* gene identified in ALS patients.

ID	Position (hg19)	Refseq ID	cDNA change	Protein change	dbSNP	Minor allele frequencies		Functional predictions									ACMG	
						gnomAD_genome_ALL	gnomAD_exome_ALL	SIFT	Polyphen2	MutationTaster	MetaSVM	MetaLR	ClinPred	M_CAP	CADD	Pathogenic (total)	Evidence	Classification
P1	chr2:241710533	NM_001330290	c.1271C>T	p.Pro424Leu	rs1254343314	–	–	–	–	–	–	–	–	–	–	–	PM2	VUS
P2	chr2:241696840	NM_001244008	c.2753_2754insGGA	p.Asp918delinsGluAsp	rs758125020	1.68E-02	1.74E-02	–	–	–	–	–	–	–	–	–	PM2, PM4	VUS
P3	chr2:241689888	NM_001244008	c.2935G>A	p.Glu979Lys	rs764324827	–	4.00E-06	T	P	D	T	D	D	D	35	6 (8)	PM2, BP4	VUS
P4	chr2:241685282	NM_001244008	c.3247G>A	p.Ala1083Thr	rs201793635	4.00E-04	3E-06	T	B	P	T	T	T	D	5.97	1 (8)	BP4	VUS
P5	chr2:241685282	NM_001244008	c.3247G>A	p.Ala1083Thr	rs201793635	4.00E-04	3.00E-04	T	B	P	T	T	T	D	5.97	1 (8)	BP4	VUS
P6	chr2:241683410	NM_001244008	c.3533 T>C	p.Phe1178Ser	-	–	–	D	D	D	–	–	–	–	25.6	3 (3)	PM2, PM1, PP3	VUS
P7	chr2:241680755	NM_001244008	c.3680C>T	p.Pro1227Leu	rs374244985	2.00E-04	2.00E-04	T	B	D	T	T	T	D	21.2	3 (8)	PM1, BP4	VUS
P8	chr2:241679768	NM_001244008	c.3763G>A	p.Val1255Met	rs752703226	–	1.20E-05	D	D	D	T	T	D	D	24.2	6 (8)	PM2, PM1, PP2, PP3	LP
P9	chr2:241661285	NM_001244008	c.4682C>T	p.Thr1561Met	rs769101887	–	4.00E-06	T	B	D	T	T	T	D	19.77	3 (8)	PM2, PM1, BP4	VUS
P10	chr2:241660421	NM_001244008	c.4778C>T	p.Pro1593Leu	rs200902828	3.00E-04	5.00E-04	D	B	D	T	T	T	D	23.6	4 (8)	PM2, PM1, PP2, PP3	LP
P11	chr2:241659285	NM_001244008	c.4927G>A	p.Asp1643Asn	rs200141437	1.00E-04	3.00E-04	T	B	D	T	T	T	T	18.6	2 (8)	PM2, PS4, PM1, PP2, BP4	LP
P12	chr2:241659257	NM_001244008	c.4955G>A	p.Arg1652Gln	rs376658420	9.70E-05	2.00E-04	T	B	P	T	T	T’	T	6.526	0 (8)	PM2, PM1, BP4	VUS
P13	chr2:241658506	NM_001244008	c.5131G>A	p.Asp1711Asn	rs199574770	3.20E-05	5.70E-05	D	D	D	T	T	T	D	34	5 (8)	PM1, PP3	VUS
P14	chr2:241658487	NM_001244008	c.5150G>T	p.Arg1717Leu	rs760970824	5.00E-05	2.40E-05	D	D	D	T	T	D	D	35	6 (8)	PM2, PM1, PP2, PP3	LP

ALS, amyotrophic lateral sclerosis; SNP, Single-nucleotide polymorphism; ACMG, American College of Medical Genetics and Genomics; VUS, uncertain significance; LP, likely pathogenic; HGMD, Human Gene Mutation Database; SIFT, sorting intolerant from tolerant; PolyPhen2, polymorphism phenotyping version 2; SVM, support vector machine; CADD, combined annotation dependent depletion; M_CAP, Mendelian clinically applicable pathogenicity; SIFT (D: Damaging; T: Tolerable); Polyphen2 (D: Probably_Damaging; P: Possibly_Damaging; B:Benign); Mutation Taster (D: Disease_causing; P: Polymorphism); MetaSVM (T: Tolerable); MetaLR (D: Damaging; T: Tolerable); ClinPred (D: Deleterious; T: Tolerable); M_CAP (D: Damaging; T: Tolerable); CADD: (D: Damaging; T: Tolerable).

In addition, 14 ALS patients with *KIF1A* mutation did not cover other ALS-related genes, including *SOD1*, *SETX*, *FUS*, *ALS2*, *OPTN*, *TARDBP*, *DCTN1*, *VAPB*, *Fig 4*, *TBK1*, *CHCHD10*, *ANXA11*, *NEK1*, *SQSTM1*, and *C9orf72*, which were classical pathogenic genes in ALS, by WES analysis. And no additional potential candidates were identified among these 14 ALS patients. In total, different nonsynonymous variants that fulfilled the same screening criteria were detected in 10 healthy controls. Ten variants (1798: p.I119T, c.356 T>C; 443: p.R355H, c.1064G>A; 1,410: p.R422C, c.1264C>T; 1,380: p.T810M, c.2429G>A; 1,158: p.D918delinsED, c.2753_2754insGGA; 568: p.E1025K, c.3073G>A; 277: p.A1083T, c.3247G>A; 1,677: p.P1227L, c.3680C>T; 1,082: p.R1296C, c.3886C>T and 1,612: p.P1688L, c.5063C>T) were detected in 1,812 healthy controls. Among them, three variants (P2: p.A918delinsED, c.2753_2754insGGA; P4, P5: p.A1083T, c.3247G>A and P7: p.P1227L, c.3680C>T), detected in ALS patients, were also found in controls. The details of these variants in *KIF1A* gene detected in controls are listed in Supplementary Table 2. In our healthy controls, the frequency of *KIF1A* variants was 0.55%. Besides, we screened the other two databases to figure out the actual frequency in Chinese population. We found the frequency was 0.49% in “gnomAD v2” database (East Asian) and 0.61% in “HUA BIAO” database, which were similar to the result in our control cohort (Supplementary Table 3).

### Regions of variants associated with ALS in the *KIF1A* gene

**Figure 1:**
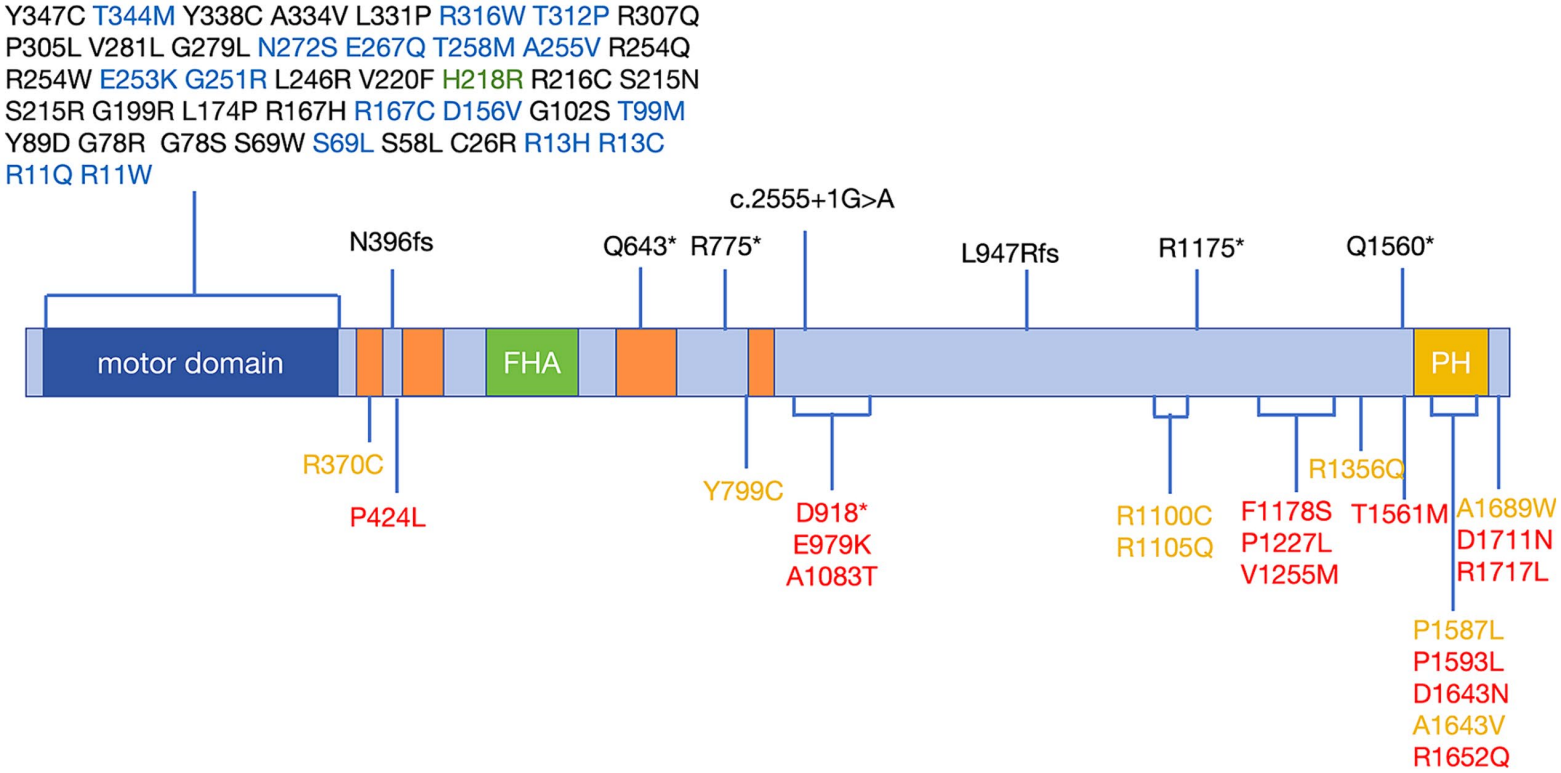
Schematic distribution of mutations in the *KIF1A* gene in KANDs. Previously reported variants associated with SPG30 and HSAN2 are listed above the schematic and are associated with SPG30 (including cases described as NESCAV syndrome) (black), HSAN2 (green), and multiple phenotypes (SPG30 and HSAN2) (blue). Variants associated with ALS are listed below the schematic and were identified in our cohort (red) and another ALS cohort (yellow). Motor domain (amino acids 5–354); CC: coiled-coil domain, CC1 (amino acids 366–383); CC2 (amino acids 429–462); CC3 (amino acids 622–681); CC4 (amino acids 801–822); FHA: Forkhead-associated domain, amino acids 516–572; and PH: pleckstrin homology domain, amino acids 1,575–1,673. Protein domains were determined according to UniProt (https://www.uniprot.org). Variants were annotated with reference to the canonical transcript NM_001244008 (p.P424L was identified in only the NM_001330290 transcript). ALS, amyotrophic lateral sclerosis; KANDs, *KIF1A*-associated neurological disorders; HSAN2, hereditary sensory and autonomic neuropathy type 2; SPG30, spastic paraplegia type 30.

*KIF1A* has been recognized as a causal gene in HSAN2, SPG30, and NESCAV syndrome. Given the overlap of clinical symptoms between these three diseases and ALS, we conducted a thorough examination of ALS patients with variations in *KIF1A* to ensure that they were not misdiagnosed. Unreported variations were detected in our patient cohort with SPG30, HSAN2, and NESCAV syndrome. To explore the correlation between *KIF1A* gene mutation and their manifestations, we examined the rare ALS-related mutations found in our research and compared them to ClinVar pathogenic variants linked to other conditions (SPG30, HSAN2, and NESCAV syndrome) (Figure 1). Specifically, mutations linked to SPG and HSAN2 were mainly found at motor domain of N-terminal of *KIF1A*, whereas those associated with ALS in our investigation and a previous investigation were primarily situated in the cargo-binding region at the C-terminal (Liao et al., 2022). Interestingly, five variants [three in our cohort and two in another ALS cohort (Liao et al., 2022)] were located in the phosphatidylinositol-binding pleckstrin homology (PH) domain.

### Genotype–phenotype correlation in patients with *KIF1A* variants

Of the 14 ALS patients with *KIF1A* mutations, two had a familial background of ALS, other 12 were sALS. The mean age of onset was 47.7 ± 12.9 years, with an age range of 23–64 years. The male-to-female ratio was 9:5. Among 14 patients, 11 were spinal-onset, while three were bulbar-onset. The mean delay in diagnosis was 35.8 ± 35.9 months. Interestingly, two patients had FTD symptoms (P3, p.E979K, c.2935G>A; and P6, p.F1178S, c.3533 T>C). Additionally, patient P6, a female, presented with repeated falls, slurred speech, and behavioral and personality changes. A neurological examination revealed that she had a masked face and a positive pull-back test result, demonstrating extrapyramidal manifestations. Interestingly, patient P10 (p.P1593L, c.4778C>T) presented with right lower limb tremor, abnormal walking gait, and progressive limb weakness. P12 walked unsteadily, had unclear articulation, and had limb weakness. Physical examination revealed increased involuntary limb movements and abnormal gait and posture. These three patients all presented extrapyramidal manifestations. Table 3 outlines the specific clinical characteristics of individuals with ALS who possess mutations in the *KIF1A* gene.

**Table 3:** Clinical features of ALS patients with mutations in *KIF1A* gene.

ID	Sex	Age at onset (years)	Site of onset	Weakness	Atrophy	Dysarthria	Dysphagia	Sensory	Reflexes	FTD symptoms	Diagnosis of delay (months)	KCSS	Survival time (months)	Family history
P1	Male	34	LL	LL	UL, LL	−	−	−	Hyper	−	12	Stage 1	135	−
P2	Male	64	LL	UL, LL	UL, LL	−	−	−	Hyper	−	9	Stage 2	85	+
P3	Male	39	G	G, UL, LL	UL	+	+	−	Hyper	+	12	Stage 3	35	−
P4	Male	23	LL	UL, LL	LL	+	−	−	Hyper	−	7	Stage 2	141	−
P5	Female	46	LL	LL	No	−	−	−	Hyper	−	77	Stage 2	>96	+
P6	Female	60	LL	LL	LL	−	−	−	Hyper	+	77	Stage 2	92	−
P7	Female	55	LL	LL	LL	−	−	−	Hyper	−	24	Stage 3	72	−
P8	Female	50	LL	UL, LL	UL, LL	−	−	−	Hypo	−	71	Stage 3	190	−
P9	Male	67	LL	UL, LL	LL	−	−	−	Hyper	−	5	Stage 1	9	−
P10	Female	51	UL	UL, LL	UL	−	−	−	Hypo	−	82	Stage 2	175	−
P11	Male	28	UL	UL, LL	UL	−	−	−	Hyper	−	12	Stage 2	32	−
P12	Male	48	G	G, UL, LL	UL, LL	+	+	−	Hypo	−	7	Stage 1	43	−
P13	Male	50	LL	LL	LL	−	−	−	Hyper	−	99	Stage 1	65	−
P14	Male	53	G	G	G	+	+	−	Hyper	−	7	Stage 1	81	−

ALS, amyotrophic lateral sclerosis; LL, lower limbs; UL, upper limbs; G, global; Hyper, hyperreflexia; Hypo, hyporeflexia; FTD, frontotemporal dementia; KCSS, King’s college staging system; “+”, affected; “−”, normal.

### Cosegregation analysis of families

**Figure 2:**
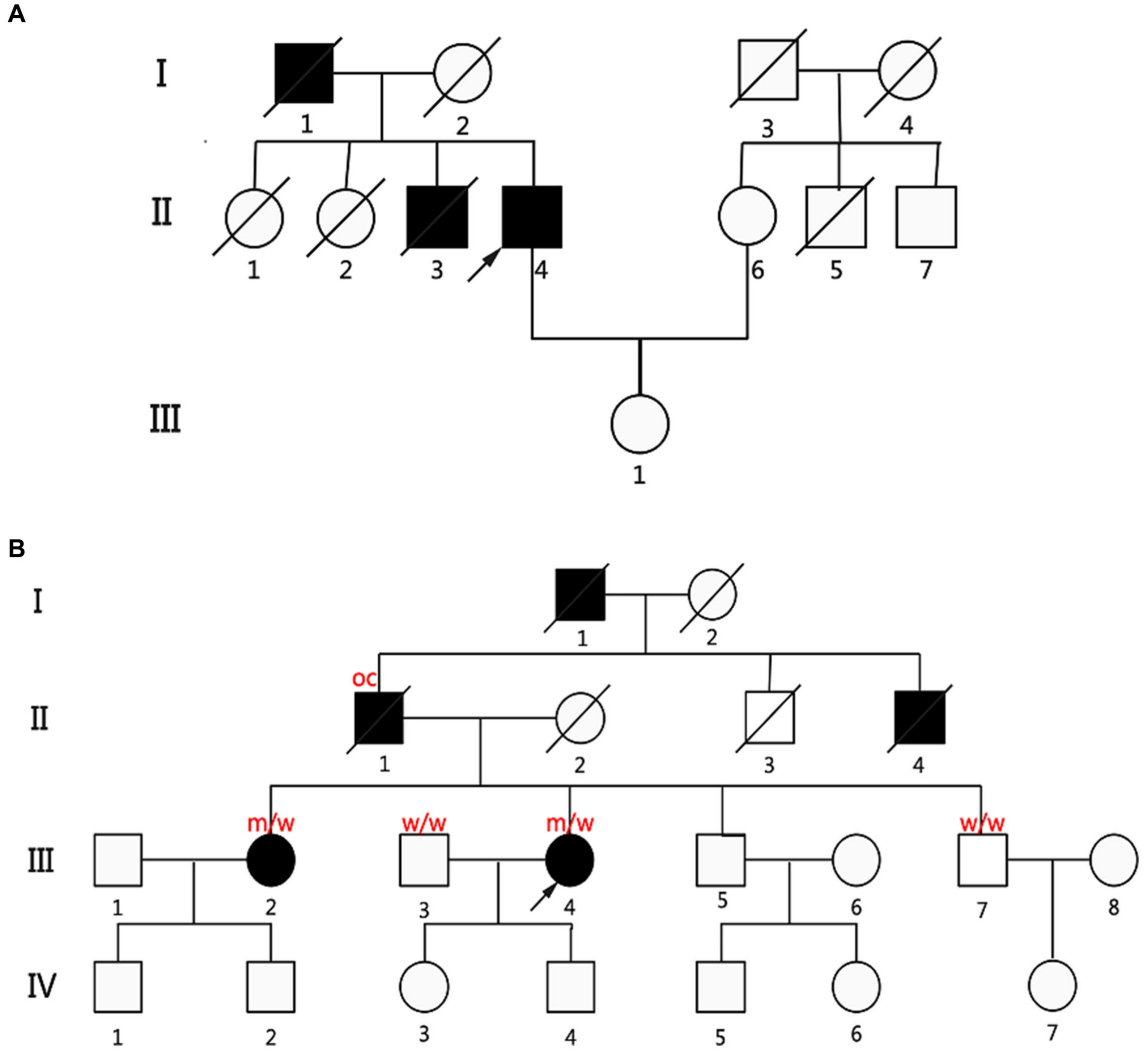
Pedigrees of two fALS patients carried the *KIF1A* missense variants. **(A)** Pedigree diagram of P2, p.A918delinsGA (c.2753_2754insGGA). **(B)** Genealogy diagram of P5, p.A1083T (c.3247G>A). Genetic analysis showing cosegregation of the *KIF1A* missense variants. Obligate carriers of the respective variant are abbreviated as “oc.” m = mutant allele; w = wild-type allele; arrow: proband; filled symbol: affected; empty symbol: unaffected; slashed symbol, decreased; square: man; circle: woman.

Next, we further corroborated the connection between missense mutations in the *KIF1A* gene and ALS through segregation analysis. Two patients (P2 and P5) had a family history. We extended the analysis to their families and reanalyzed them using Sanger sequencing (Figure 2). P2 presented with lower-limb onset and late disease onset. As shown in Supplementary Table 4, in the P2 family, the patient’s father (I:1) and older brother (II:4) presented with lower-limb onset, and upper and lower limbs muscle atrophy and weakness, similar to P2’s symptoms, and were diagnosed with ALS. Both his father and older brother had an earlier onset age, and more longer survival time than him, showing a slower progression. Detailed clinical features of ALS patients in families of P2 were shown in Supplementary Table 4. Unfortunately, the father, brother, and sisters of the proband had all passed away prior to the study, resulting in a lack of available DNA samples for cosegregation analysis.

Patient 2 also had two older sisters (II:1 and II:2; Figure 2A) who did not exhibit any ALS symptoms during their lifetime. However, unfortunately, his father, brother, and sisters had all died before the time of the study; thus, no DNA samples were available for cosegregation. The other patient (P5 and III:4; Figure 2B) with a family history had an early disease onset (46 years) and a long diagnostic delay (77 months). Four of P5’s relatives were diagnosed with ALS or self-reported symptoms consistent with ALS (father (II:1), grandfather (I:1), uncle (II:4), and older sister (III:2); Figure 2B). P5’s father and older sister had symptoms similar to hers. Her grandfather and uncle had muscle atrophy and weakness before they died. The *KIF1A* variant identified from P5 was assessed in her older sister, younger brother, and husband. Interestingly, her older sister carried the same *KIF1A* variant, while her younger brother did not. It could not be determined whether her father carried the loss-of-function mutation. Besides ALS, we did not find “other related” disorders running in the families of 14 ALS patients carried *KIF1A* mutation, such as frontotemporal dementia, cervical spondylosis, syringomyelia, peripheral neuropathy, Parkinson’s disease, and Alzheimer’s disease.

## Discussion

Thirteen variants of the *KIF1A* gene were detected in 14 of 1,068 ALS patients, resulting in a frequency of 1.31% (14/1,068) in *KIF1A*. This frequency aligns with the mutation frequency observed in a research conducted in southern China (Liao et al., 2022), which revealed a frequency of 1.06% (10/941). Our study represents the largest cohort of ALS patients with mutations in the *KIF1A* gene to date. Our research revealed 11 novel mutation variants in the *KIF1A* gene linked to ALS. Four different mutations [p.E979K (c.2935G>A, P3), p.V1255M (c.3763G>A, P8), p.D1711N (c.5131G>A, P13), and p.R1717L (c.5150G>T, P14)] were identified as potentially harmful by a combination of 5–6 computational tools. Additionally, four of 14 mutations [p.V1255M (c.3763G>A, P8), p.P1593L (c.4778C>T, P10), p.D1643N (c.4927G>A, P11), and p.R1717L (c.5150G>T, P14)] were identified to be pathogenic according to the ACMG recommendations and software prediction results, highlighting the significance of the *KIF1A* gene as a potential genetic determinant of ALS. Additionally, we did not find 10 controls who were detected *KIF1A* mutations have any obvious neurological impairment. In Chinese population, the frequencies was 0.49–0.61% based on our healthy controls (1,812 controls) and other database (11,708 controls). Consistent with the connection between *KIF1A* and ALS, the missense mutation p.A1083T (c.3247G>A) was shown to cosegregate with the disease. Although lack of available DNA samples for cosegregation analysis, we found three patients in families of P2 all presented with lower-limb onset and late disease onset, via reviewing their medical records to gather additional clinical history and neurological examination data.

This study revealed high clinical heterogeneity among ALS individuals harboring *KIF1A* gene missense mutations. Disease onset age spanned from 23 to 64 years, while the diagnostic delay varied from 5 to 99 months. The research demonstrated significant clinical diversity. Interestingly, several genotype–phenotype correlations were noted. Among the 13 ALS patients harboring the *KIF1A* gene, three had onset in the bulbar region, eight had onset in the upper limbs, and only two had onset in the lower limbs. Additionally, extrapyramidal symptoms were observed in three of these ALS patients, suggesting that upper limb onset and extrapyramidal manifestations may be characteristic of the ALS phenotype caused by the *KIF1A* gene; however, additional evidence is required. Unlike the patient cohort in a prior study conducted in China (Liao et al., 2022), our cohort of ALS patients harboring *KIF1A* missense mutations did not exhibit obvious sensory impairment, highlighting the high clinical heterogeneity of ALS patients harboring *KIF1A* variants.

*KIF1A* and *KIF5A* are KIFs that function as molecular motors, utilizing chemical energy from ATPs to transport cargo along microtubules. Studies have indicated that the mutation frequency of *KIF5A* in the Chinese sALS population ranges from 0.16% (1/645) (Zhang et al., 2019) to 0.41% (2/581) (Gu et al., 2019; He et al., 2020). In the Western population, the mutation frequency of *KIF5A* is reported to be 0.47–0.53% (Brenner et al., 2018; Nicolas et al., 2018), which is greater than that in the Chinese population. Another study of the Norwegian population revealed that *KIF1A* risk variants were present in 1.08% (3/279) of ALS patients, consistent with the findings in the Chinese population (Olsen et al., 2024). These results demonstrate that *KIF1A* is a more prevalent ALS-associated gene than *KIF5A* in the Chinese population. *KIF5A* is a more frequent determinant of ALS in the European population, while *KIF1A* accounts for a similar proportion of ALS patients in European and Chinese populations.

There is genetic overlap among SPG, HSAN2, and ALS. For example, SPG11 and *KIF5A* have been found to be pathogenic in both ALS and SPG (Stevanin et al., 2007; Orlacchio et al., 2010; Nicolas et al., 2018), and *SPTLC1* has been recognized as a novel risk gene factor in ALS and HSAN2 (Lone et al., 2022). Our latest discoveries and prior investigations indicate that *KIF1A* might potentially serve as a shared causative gene linked to SPG, HSAN2, and ALS. Based on this, we posit that SPG, HSAN2, and ALS may represent a range of characteristics linked to variations in the *KIF1A* gene. Similar to findings associated with *KIF5A*, we have identified varying mutation distributions in *KIF1A* across different diseases. Specifically, in HSP/Charcot-Marie-Tooth 2 patients, the majority of *KIF5A* mutations are situated in the motor domain, whereas ALS patients tend to have mutations in the C-terminal cargo-binding domain. Mutations in *KIF1A* linked to SPG and HSAN2 mainly occurred in the motor domain at the N-terminal, while alterations associated with ALS, as indicated by our study and corroborated by prior research, were mainly found in the cargo-binding region at the C-terminal (Liao et al., 2022). It could be speculated that *KIF1A* and *KIF5A* mutations tend to lead to the ALS phenotype when the C-terminal cargo-binding region is influenced and hereditary peripheral neuropathy and the HSP phenotype when the N-terminal motor domain is influenced. The clinical manifestations of *KIF1A*-related neuropathy disorders vary widely, with *KIF1A* being the common cause. As a result, these conditions are classified as “*KIF1A*-associated neurological disorders (KAND)” (Boyle et al., 2021). Differences in gene function may cause the diversity of clinical phenotypes.

## Conclusion

In conclusion, we demonstrated that pathogenic *KIF1A* variants were associated with ALS and analyzed the genotype–phenotype correlation of patients with *KIF1A* variants. Our finding widened the genotypic spectrum of *KIF1A* and supplement prior findings of *KIF1A*-related ALS.

## Data availability statement

The datasets presented in this study can be found in the article/Supplementary material.

## Ethics statement

The studies involving humans were approved by the Institutional Ethics Committee of PUTH, IRB00006761. The studies were conducted in accordance with the local legislation and institutional requirements. The participants provided their written informed consent to participate in this study.

## Author contributions

WZ: Conceptualization, Methodology, Writing – original draft. JH: Writing – review & editing. LC: Investigation, Writing – review & editing. WY: Resources, Writing – review & editing. NZ: Validation, Writing – review & editing. XL: Funding acquisition, Project administration, Supervision, Writing – review & editing. DF: Funding acquisition, Project administration, Supervision, Writing – review & editing.

## Footnotes

^1^http://bio-bwa.sourceforge.net/

^2^http://www.broadinstitute.org/gatk

^3^http://www.mutationtaster.org

^4^http://sift.jcvi.org/

^5^http://genetics.bwh.harvard.edu/pph2/

^6^https://sites.google.com/site/clinpred/

^7^http://bejerano.stanford.edu/mcap/

^8^https://cadd.gs.washington.edu

## References

[ref1] Anazawa Y. Kita T. Iguchi R. Hayashi K. Niwa S. (2022). De novo mutations in KIF1A-associated neuronal disorder (KAND) dominant-negatively inhibit motor activity and axonal transport of synaptic vesicle precursors. Proc. Natl. Acad. Sci. U. S. A. 119:e2113795119. doi: 10.1073/pnas.2113795119, PMID: 35917346 PMC9371658

[ref2] Baldwin K. R. Godena V. K. Hewitt V. L. Whitworth A. J. (2016). Axonal transport defects are a common phenotype in Drosophila models of ALS. Hum. Mol. Genet. 25, 2378–2392. doi: 10.1093/hmg/ddw105, PMID: 27056981 PMC5181624

[ref3] Bilsland L. G. Sahai E. Kelly G. Golding M. Greensmith L. Schiavo G. (2010). Deficits in axonal transport precede ALS symptoms in vivo. Proc. Natl. Acad. Sci. U. S. A. 107, 20523–20528. doi: 10.1073/pnas.1006869107, PMID: 21059924 PMC2996651

[ref4] Boyle L. Rao L. Kaur S. Fan X. Mebane C. Hamm L.. (2021). Genotype and defects in microtubule-based motility correlate with clinical severity in KIF1A-associated neurological disorder. HGG Adv. 2:100026. doi: 10.1016/j.xhgg.2021.100026 33880452 PMC8054982

[ref5] Brenner D. Freischmidt A. (2022). Update on genetics of amyotrophic lateral sclerosis. Curr. Opin. Neurol. 35, 672–677. doi: 10.1097/WCO.0000000000001093 35942673

[ref6] Brenner D. Yilmaz R. Muller K. Grehl T. Petri S. Meyer T.. (2018). Hot-spot KIF5A mutations cause familial ALS. Brain 141, 688–697. doi: 10.1093/brain/awx370, PMID: 29342275 PMC5837483

[ref7] Brooks B. R. Miller R. G. Swash M. Munsat T. L. World Federation of Neurology Research Group on Motor Neuron Diseases (2000). El Escorial revisited: revised criteria for the diagnosis of amyotrophic lateral sclerosis. Amyotroph. Lateral Scler. Other Motor Neuron Disord. 1, 293–299. doi: 10.1080/146608200300079536 11464847

[ref8] Brown R. H. Al-Chalabi A. (2017). Amyotrophic lateral sclerosis. N. Engl. J. Med. 377, 162–172. doi: 10.1056/NEJMra1603471 28700839

[ref9] Castellanos-Montiel M. J. Chaineau M. Durcan T. M. (2020). The neglected genes of ALS: cytoskeletal dynamics impact synaptic degeneration in ALS. Front. Cell. Neurosci. 14:594975. doi: 10.3389/fncel.2020.594975, PMID: 33281562 PMC7691654

[ref10] Chia R. Chio A. Traynor B. J. (2018). Novel genes associated with amyotrophic lateral sclerosis: diagnostic and clinical implications. Lancet Neurol. 17, 94–102. doi: 10.1016/S1474-4422(17)30401-5, PMID: 29154141 PMC5901717

[ref11] Edwards S. L. Yorks R. M. Morrison L. M. Hoover C. M. Miller K. G. (2015). Synapse-assembly proteins maintain synaptic vesicle cluster stability and regulate synaptic vesicle transport in *Caenorhabditis elegans*. Genetics 201, 91–116. doi: 10.1534/genetics.115.177337, PMID: 26354975 PMC4566279

[ref12] Feldman E. L. Goutman S. A. Petri S. Mazzini L. Savelieff M. G. Shaw P. J.. (2022). Amyotrophic lateral sclerosis. Lancet 400, 1363–1380. doi: 10.1016/S0140-6736(22)01272-7, PMID: 36116464 PMC10089700

[ref13] Fischer L. R. Culver D. G. Tennant P. Davis A. A. Wang M. Castellano-Sanchez A.. (2004). Amyotrophic lateral sclerosis is a distal axonopathy: evidence in mice and man. Exp. Neurol. 185, 232–240. doi: 10.1016/j.expneurol.2003.10.004, PMID: 14736504

[ref14] Goutman S. A. Hardiman O. Al-Chalabi A. Chio A. Savelieff M. G. Kiernan M. C.. (2022a). Emerging insights into the complex genetics and pathophysiology of amyotrophic lateral sclerosis. Lancet Neurol. 21, 465–479. doi: 10.1016/S1474-4422(21)00414-2, PMID: 35334234 PMC9513754

[ref15] Goutman S. A. Hardiman O. Al-Chalabi A. Chio A. Savelieff M. G. Kiernan M. C.. (2022b). Recent advances in the diagnosis and prognosis of amyotrophic lateral sclerosis. Lancet Neurol. 21, 480–493. doi: 10.1016/S1474-4422(21)00465-8, PMID: 35334233 PMC9513753

[ref16] Gu X. Li C. Chen Y. Wei Q. Cao B. Ou R.. (2019). Mutation screening of the KIF5A gene in Chinese patients with amyotrophic lateral sclerosis. J. Neurol. Neurosurg. Psychiatry 90, 245–246. doi: 10.1136/jnnp-2018-318395 29954873

[ref17] Guedes-Dias P. Nirschl J. J. Abreu N. Tokito M. K. Janke C. Magiera M. M.. (2019). Kinesin-3 responds to local microtubule dynamics to target synaptic cargo delivery to the presynapse. Curr. Biol. 29:e268, 268–282.e8. doi: 10.1016/j.cub.2018.11.065, PMID: 30612907 PMC6342647

[ref18] Guo W. Naujock M. Fumagalli L. Vandoorne T. Baatsen P. Boon R.. (2017). HDAC6 inhibition reverses axonal transport defects in motor neurons derived from FUS-ALS patients. Nat. Commun. 8:861. doi: 10.1038/s41467-017-00911-y, PMID: 29021520 PMC5636840

[ref19] He J. Liu X. Tang L. Zhao C. He J. Fan D. (2020). Whole-exome sequencing identified novel KIF5A mutations in Chinese patients with amyotrophic lateral sclerosis and Charcot-Marie-Tooth type 2. J. Neurol. Neurosurg. Psychiatry 91, 326–328. doi: 10.1136/jnnp-2019-320483, PMID: 31422367

[ref20] Hirokawa N. Noda Y. Tanaka Y. Niwa S. (2009). Kinesin superfamily motor proteins and intracellular transport. Nat. Rev. Mol. Cell Biol. 10, 682–696. doi: 10.1038/nrm2774 19773780

[ref21] Liao P. Yuan Y. Liu Z. Hou X. Li W. Wen J.. (2022). Association of variants in the KIF1A gene with amyotrophic lateral sclerosis. Transl. Neurodegener. 11:46. doi: 10.1186/s40035-022-00320-2, PMID: 36284339 PMC9597953

[ref22] Liu X. He J. Chen L. Zhang N. Tang L. Liu X.. (2021). TBK1 variants in Chinese patients with amyotrophic lateral sclerosis. Neurobiol. Aging 97, 149.e9–149.e15. doi: 10.1016/j.neurobiolaging.2020.07.028 32893041

[ref23] Liu X. Yang L. Tang L. Chen L. Liu X. Fan D. (2017). DCTN1 gene analysis in Chinese patients with sporadic amyotrophic lateral sclerosis. PLoS One 12:e0182572. doi: 10.1371/journal.pone.0182572, PMID: 28792508 PMC5549744

[ref24] Lone M. A. Aaltonen M. J. Zidell A. Pedro H. F. Morales Saute J. A. Mathew S.. (2022). SPTLC1 variants associated with ALS produce distinct sphingolipid signatures through impaired interaction with ORMDL proteins. J. Clin. Invest. 132:e161908. doi: 10.1172/JCI161908, PMID: 35900868 PMC9479574

[ref25] Mckenna A. Hanna M. Banks E. Sivachenko A. Cibulskis K. Kernytsky A.. (2010). The genome analysis toolkit: a MapReduce framework for analyzing next-generation DNA sequencing data. Genome Res. 20, 1297–1303. doi: 10.1101/gr.107524.110, PMID: 20644199 PMC2928508

[ref26] Nicita F. Ginevrino M. Travaglini L. D'arrigo S. Zorzi G. Borgatti R.. (2021). Heterozygous KIF1A variants underlie a wide spectrum of neurodevelopmental and neurodegenerative disorders. J. Med. Genet. 58, 475–483. doi: 10.1136/jmedgenet-2020-107007, PMID: 32737135

[ref27] Nicolas A. Kenna K. P. Renton A. E. Ticozzi N. Faghri F. Chia R.. (2018). Genome-wide analyses identify KIF5A as a novel ALS gene. Neuron 97:e1266. doi: 10.1016/j.neuron.2018.02.027 PMC5867896 29566793

[ref28] Olsen C. G. Busk O. L. Holla O. L. Tveten K. Holmoy T. Tysnes O. B.. (2024). Genetic overlap between ALS and other neurodegenerative or neuromuscular disorders. Amyotroph. Lateral Scler. Frontotemporal. Degener. 25, 177–187. doi: 10.1080/21678421.2023.2270705 37849306

[ref29] Orlacchio A. Babalini C. Borreca A. Patrono C. Massa R. Basaran S.. (2010). SPATACSIN mutations cause autosomal recessive juvenile amyotrophic lateral sclerosis. Brain 133, 591–598. doi: 10.1093/brain/awp325, PMID: 20110243 PMC2822627

[ref30] Stevanin G. Santorelli F. M. Azzedine H. Coutinho P. Chomilier J. Denora P. S.. (2007). Mutations in SPG11, encoding spatacsin, are a major cause of spastic paraplegia with thin corpus callosum. Nat. Genet. 39, 366–372. doi: 10.1038/ng1980 17322883

[ref31] Stucchi R. Plucińska G. Hummel J. J. A. Zahavi E. E. Guerra San Juan I. Klykov O.. (2018). Regulation of KIF1A-driven dense core vesicle transport: ca(2+)/CaM controls DCV binding and Liprin-alpha/TANC2 recruits DCVs to postsynaptic sites. Cell Rep. 24, 685–700. doi: 10.1016/j.celrep.2018.06.071, PMID: 30021165 PMC6077247

[ref32] Tanaka Y. Niwa S. Dong M. Farkhondeh A. Wang L. Zhou R.. (2016). The molecular motor KIF1A transports the TrkA neurotrophin receptor and is essential for sensory neuron survival and function. Neuron 90, 1215–1229. doi: 10.1016/j.neuron.2016.05.002, PMID: 27263974

[ref33] Tang L. Chen L. Liu X. He J. Ma Y. Zhang N.. (2022). The repeat length of C9orf72 is associated with the survival of amyotrophic lateral sclerosis patients without C9orf72 pathological expansions. Front. Neurol. 13:939775. doi: 10.3389/fneur.2022.939775, PMID: 35989899 PMC9381700

[ref34] Zhang K. Liu Q. Shen D. Tai H. Liu S. Wang Z.. (2019). Mutation analysis of KIF5A in Chinese amyotrophic lateral sclerosis patients. Neurobiol. Aging 73, 229.e1–229.e4. doi: 10.1016/j.neurobiolaging.2018.08.006 30301576

