# Supplementary material for: Genetic link between KIF1A mutations and amyotrophic lateral sclerosis: evidence from whole-exome sequencing
Source: Front Aging Neurosci. 2024 Jul 15;16:1421841. doi: 10.3389/fnagi.2024.1421841 (PMC11284166; doi:10.3389/fnagi.2024.1421841)
Supplement: Supplementary file 1 [file Table_1.docx]

**Supplementary Table 1. Clinical comorbidities/diseases of controls in this study**

|  | Number | Percent |
| --- | --- | --- |
| Hypertension | 264 | 14.57% |
| Coronary heart disease | 308 | 17.00% |
| Diabetes (without peripheral neuropathy) | 352 | 19.43% |
| Tumor | 168 | 9.27% |
| Chronic obstructive pulmonary disease | 132 | 7.28% |

**Supplementary Table 2. Details of variants in the *KIF1A* gene identified in controls**

| ID | Chromosomal Position (hg19) | Refseq ID | cDNA change | Protein change | Het/Hom | dbSNP | gnomAD_genome_ALL | gnome AD_exome_ALL |
| --- | --- | --- | --- | --- | --- | --- | --- | --- |
| 1798 | chr2: 241727475 | NM_001244008 | c.356T>C | p.Ile119Thr | Het | rs1482769652 | / | 2.00E-04 |
| 443 | chr2: 241712647 | NM_001244008 | c.1064G>A | p.Arg355His | Het | rs373042822 | / | 2.84E-05 |
| 1410 | chr2: 241710465 | NM_001244008 | c.1264C>T | p.Arg422Cys | Het | rs374011613 | / | 2.03E-05 |
| 1380 | chr2:241700097 | NM_001244008 | c.2429C>T | p.Thr810Met | Het | rs772046874 | / | 2.06E-05 |
| 1158 | chr2: 241696840 | NM_001244008 | c.2753_2754insGGA | p.Asp918delinsGluAsp | Het | rs758125020 | 1.68E-02 | 1.74E-02 |
| 568 | chr2: 241685585 | NM_001244008 | c.3073G>A | p.Glu1025Lys | Het | rs1476899818 | / | 1.82E-05 |
| 277 | chr2: 241685282 | NM_001244008 | c.3247G>A | p.Ala1083Thr | Het | rs201793635 | 4.00E-04 | 3.00E-04 |
| 1677 | chr2: 241680755 | NM_001244008 | c.3680C>T | p.Pro1227Leu | Het | rs374244985 | 2.00E-04 | 2.00E-04 |
| 1082 | chr2: 241679490 | NM_001244008 | c.3886C>T | p.Arg1296Cys | Het | rs201684653 | 6.46E-05 | 2.95E-05 |
| 1612 | chr2: 241658574 | NM_001244008 | c.5063C>T | p.Pro1688Leu | Het | rs549560429 | 6.47E-05 | 2.87E-05 |

Chr: chromosome; Het: heterozygous; Hom: Homozygous; SNP: single nucleotide polymorphism

**Supplementary Table 3. Frequencies of *KIF1A* in Chinese populations based on different database**

|  | Total number | Frequencies |
| --- | --- | --- |
| Our healthy controls | 1812 | 0.55% |
| gnomAD v2 database (East Asian) | 6708 | 0.49% |
| HUA BIAO database | 5000 | 0.61% |

**Supplementary Table 4. Clinical features of ALS patients in families of P2**

| Patient | Sex | Age at onset (years) | Site of onset | Weakness | Atrophy | Dysarthria | Dysphagia | Sensory | Reflexes | FTD symptoms | Diagnosis of delay (months) | KCSS | Survival time (months) |
| --- | --- | --- | --- | --- | --- | --- | --- | --- | --- | --- | --- | --- | --- |
| II-4 (Proband) | male | 55 | LL | G, UL, LL | UL, LL | + | + | - | Hyper | - | 11 | Stage 3 | 85 |
| II-1 (Father) | male | 49 | LL | UL, LL | UL, LL | - | - | - | Hyper | - | 18 | Stage 2 | 102 |
| II-3 (Brother) | male | 43 | LL | G, UL, LL | G, UL, LL | + | + | - | Hyper | - | 16 | Stage 3 | 135 |

ALS: amyotrophic lateral sclerosis; LL: lower limbs; UL: upper limbs; G: global; Hyper: hyperreflexia; Hypo: hyporeflexia; FTD: frontotemporal dementia; KCSS: King’s college staging system; “+”: affected; “-”: normal.
